# Ionic Transport Properties of Cation-Exchange Membranes Prepared from Poly(vinyl alcohol-*b*-sodium Styrene Sulfonate)

**DOI:** 10.3390/membranes11060452

**Published:** 2021-06-19

**Authors:** Yuriko Kakihana, N. Awanis Hashim, Taiko Mizuno, Marika Anno, Mitsuru Higa

**Affiliations:** 1 Graduate School of Sciences and Technology for Innovation, Yamaguchi University, 2-16-1 Tokiwadai, Ube Yamaguchi 755-8611, Japan; kakihana@yamaguchi-u.ac.jp (Y.K.); mtaiko@outlook.jp (T.M.); a066vfu@yamaguchi-u.ac.jp (M.A.); 2 Blue Energy Center for SGE Technology (BEST), 2-16-1 Tokiwadai, Ube City, Yamaguchi 755-8611, Japan; 3 Department of Chemical Engineering, Faculty of Engineering, Universiti Malaya, Kuala Lumpur 50603, Malaysia; awanis@um.edu.my

**Keywords:** poly(vinyl alcohol), block copolymer, membrane resistance, transport number, cation-exchange membrane

## Abstract

Membrane resistance and permselectivity for counter-ions have important roles in determining the performance of cation-exchange membranes (CEMs). In this study, PVA-based polyanions—poly(vinyl alcohol-*b*-sodium styrene sulfonate)—were synthesized, changing the molar percentages *C*_CEG_ of the cation-exchange groups with respect to the vinyl alcohol groups. From the block copolymer, poly(vinyl alcohol) (PVA)-based CEMs, hereafter called “B-CEMs”, were prepared by crosslinking the PVA chains with glutaraldehyde (GA) solution at various GA concentrations *C*_GA_. The ionic transport properties of the B-CEMs were compared with those previously reported for the CEMs prepared using a random copolymer—poly(vinyl alcohol-co-2-acrylamido-2-methylpropane sulfonic acid)—hereafter called ”R-CEMs”. The B-CEMs had lower water content than the R-CEMs at equal molar percentages of the cation-exchange groups. The charge density of the B-CEMs increased as *C*_CEG_ increased, and reached a maximum value, which increased with increasing *C*_GA_. A maximum charge density of 1.47 mol/dm^3^ was obtained for a B-CEM with *C*_CEG_ = 2.9 mol% and *C*_GA_ = 0.10 vol.%, indicating that the B-CEM had almost two-thirds of the permselectivity of a commercial CEM (CMX: ASTOM Corp. Japan). The dynamic transport number and membrane resistance of a B-CEM with *C*_CEG_ = 8.3 mol% and *C*_GA_ = 0.10 vol.% were 0.99 and 1.6 Ωcm^2^, respectively. The B-CEM showed higher dynamic transport numbers than those of the R-CEMs with similar membrane resistances.

## 1. Introduction

In recent years, cation-exchange membranes (CEMs) have received considerable attention from researchers and manufacturers, and have been widely used for various industrial purposes [1], such as the separation of metal ion pollutants from hard water [2], chlor-alkali electrolysis [3], fuel cells [4,5,6,7,8,9], electrodialytic concentration or desalination of electrolyte solutions [1,3,10], redox flow batteries [11,12,13], reverse electrodialysis [14,15,16], and hydrogen production [17,18,19]. CEMs contain fixed, negatively charged groups that allow cations to pass through the membranes while concurrently rejecting anions. Most commercially available CEMs for electrodialysis are made of styrene-co-divinylbenzene matrices or other conjugated polymers. However, the use of this type of membrane is limited by difficulties in controlling the membrane structure due to simultaneous copolymerization and crosslinking. Furthermore, the cost of manufacturing CEMs is relatively high. Therefore, many novel CEMs have been developed recently to overcome these problems [20,21,22]. These CEMs offer better control over the distribution of the fixed charges, and exhibit similar performance to conventional cation-exchange membranes. Some ion-exchange membranes (IEMs) are prepared via the combination of water-soluble base polymers and polyelectrolytes, followed by crosslinking of the base polymers. The obtained IEM has a semi-interpenetrating network (semi-IPN) structure, meaning that the polyelectrolyte chains are fixed in a crosslinked polymer matrix. The ion-exchange capacity of IEMs thus obtained can be easily controlled via changes to the mixture ratio of the base polymer to the polyelectrolyte. One widely used water-soluble base polymer—poly(vinyl alcohol) (PVA)—is a polyhydroxy polymer that has favorable physicochemical properties [23,24,25,26] for use as a base polymer in IEMs. PVA has a low methanol permeability; hence, there have been many reports of PVA-based CEMs for direct methanol fuel cell applications [27,28,29,30,31,32,33]. Various types of PVA-based CEMs with semi-IPN structures have been prepared; however, the stability of such CEMs in aqueous solutions is a potential disadvantage. To overcome this disadvantage, CEMs with IPN structures have been prepared from a mixture of PVA and PVA-based polyanions [34,35,36,37]. CEMs with IPN structures consist of crosslinked polymers in which negatively charged groups are fixed covalently; hence, they show higher long-term aqueous stability than CEMs with semi-IPN structures. However, CEMs with IPN structures prepared from a random copolymer—poly(vinyl alcohol-co-2-acrylamido-2-methylpropane sulfonic acid [37]—hereafter called “R-CEMs”, showed much lower transport properties than commercially available CEMs.

**Figure 1:**
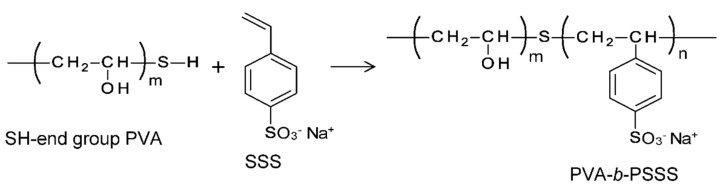
Chemical structure of SH end group PVA, SSA, and PVA-*b*-PSSS, and reaction scheme of PVA-*b*-PSSS.

The aim of this study was to prepare a PVA-based CEM with superior higher counter-ion transport properties to R-CEMs. To this end, samples of a block copolymer of vinyl alcohol and sodium *p*-styrenesulfonate groups (hereafter referred to as PVA-*b*-PSSS) with various ratios of cation-exchange groups were polymerized. CEMs with IPN structures were prepared from the block copolymer, hereafter called “B-CEMs”. Figure 1 shows the chemical structure of the PVA-*b*-PSSS. The crystalline region of the semicrystalline PVA acts as a physical crosslink point. In a film-forming process involving the PVA-based block copolymer, microphase separation of the PVA phase and the charged phase occurs, while heat treatment increases the crystallinity of the PVA region. During this process, the charged groups are concentrated into amorphous regions, resulting in the formation of ionic paths with high charge densities in the cation-exchange groups [33,38]. Therefore, the B-CEMs have a higher cation selectivity and lower membrane resistance than those of CEMs with low charge densities. The crystallinity of the PVA region within a B-CEM is higher than that of an R-CEM, because the cation-exchange groups in the random copolymer chains inhibit the formation of crystals. Thus, the B-CEMs have a higher counter-ion permselectivity than the R-CEMs. 

In this study, we investigated the differences in the ionic transport properties—i.e., charge density, membrane resistance, and transport number—of PVA-based CEMs prepared from two different copolymers.

## 2. Experimental Methods

### 2.1. Materials 

SH end group PVA (PVA-SH: 100% hydrolyzed, average Mw = 198,000) was obtained from Kuraray Co., Ltd., Tokyo, Japan. Sodium p-styrenesulfonate (SSS) was purchased from Tosoh Co., Tokyo, Japan. 2,2′-Azobis(2-methylpropionamidine) dihydrochloride (V-50) and analytical grade glutaraldehyde (GA) (25 wt.% solution in water) were purchased from Wako Pure Chemical Industries, Osaka, Japan. Sodium chloride, hydrochloric acid, and potassium chloride (all analytical grade) were purchased from Nacalai Tesque, Kyoto, Japan.

### 2.2. Synthesis of Block Copolymers 

PVA-SH was dissolved in deionized water at 90 °C. Various amounts of SSS were then added to different batches of the solution in order to change the SSS content of the polymer. The mixed aqueous solutions of PVA-SH and SSS were purged with nitrogen gas and stirred for 30 min before the initiator (V-50) was gradually added over 2 h, after which the reaction was allowed to proceed for 1.5 h at 90 °C [39]. After polymerization, the unreacted monomer and homopolymer (polySSS) were removed by precipitation in acetone. The block copolymers were then dried under vacuum at 50 °C to a constant weight. The reaction conditions for the block copolymers are listed in Table 1.

### 2.3. Analysis of Chemical Structure of Block Copolymers

^1^H nuclear magnetic resonance (NMR) spectra of the PVA-SH, SSS monomer, and block copolymers were obtained using an NMR spectrometer (JEOL JNM-EX270FT NMR system). Dimethyl sulfoxide (DMSO)-d_6_ and D_2_O were used as the solvents, and tetramethylsilane was used as the internal standard. The data were analyzed using commercially available software (JEOL DATYM LTD. ALICE2) to estimate the SSS content of the block copolymers.

### 2.4. Preparation of B-CEMs 

Aqueous solutions of PVA-*b*-PSSS with various SSS contents were cast on an acrylic plate and dried over a hot stage (NISSIN, NH-45N) for 24 h overnight at 50 °C. Self-standing base films of the B-CEMs were obtained and annealed under vacuum at 160 °C for 30 min to induce physical crosslinking between the PVA chains. The annealed films were immersed in aqueous solutions containing GA, whose concentrations are shown in Table 2, along with 0.05 mol/dm^3^ of HCl and 3.0 mol/dm^3^ NaCl, at 25 °C for 24 h, to facilitate chemical crosslinking. The resulting B-CEMs were kept in 0.1 mol/dm^3^ NaCl solution in order to maintain an equilibrium swelling state. 

### 2.5. Measurement of Membrane Water Content

To measure the membrane water content *H*, the membrane was removed from the 0.1 mol/dm^3^ NaCl solution, dabbed with filter paper to remove excess water on the membrane surfaces, and the weight of the wet sample membrane immersed in NaCl solution (the counter-ion of the membrane was Na^+^) *W*_w_ was measured. The value of the weight in a dry state *W*_d_ measured at the ion-exchange capacity (*IEC*) was used. The volumetric water content was calculated from *W*_w_ and *W*_d_ as follows:

(1) $$\Delta H\equiv \frac{\left(W_{w}-W_{d}\right)/1.0}{\left(W_{w}-W_{d}\right)/1.0+\frac{W_{d}}{1.3}}$$

where 1.0 and 1.3 are the densities of water and PVA [40], respectively. 

### 2.6. Measurement of Ion-Exchange Capacity (IEC)

*IEC* is defined as the milliequivalent of cation-exchange groups per 1 g of dry membrane whose counter-ions are Na^+^ ions (meq/g-dry-Na form). To measure the *IEC* of a sample membrane, the membrane of 5 cm × 5 cm was immersed in 0.5 mol/dm^3^ KCl solution for 6 h to change the counter-ions with K^+^ ions. The membrane was then immersed in 50 cm^3^ of 0.3 mol/dm^3^ of NaNO_3_ and stirred for 24 h to ensure that all of the K^+^ counter-ions were exchanged with the Na^+^ ions in the solution. The concentration of K^+^ ions C_K+_ in the solution was measured using an ion chromatograph (Dionex ICS-1500). The membrane was dried under vacuum for 24 h, and its dry weight *W*_d_ was measured. The *IEC* of the membrane was obtained using the following equation:

(2) $$IEC=\frac{C_{K+}}{W_{d}}\times \frac{100}{1000}$$

### 2.7. Determination of Membrane Charge Density

To estimate the charge density of the membrane, the membrane potential Δφ was measured at 25.0 °C ± 0.5 °C in a diffusion dialysis system with two KCl solutions at different concentrations *C*_o_ and *C*_d_ (r = *C*_d_/*C*_o_ = 5). The membrane charge density *C*_x_ was calculated from the relationship between Δφ and *C*_o_ using the following equation [41]:

(3) $$\Delta \emptyset =-\frac{RT}{F}\ln\left(r\cdot \frac{\sqrt{C_{x}^{2}+{\left(2C_{0}\right)}^{2}}-C_{x}}{\sqrt{C_{x}^{2}+{\left(2rC_{0}\right)}^{2}}-C_{x}}\right)-\frac{RT}{F}W\ln\left(\frac{\sqrt{C_{x}^{2}+{\left(2rC_{0}\right)}^{2}}-C_{x}W}{\sqrt{C_{x}^{2}+{\left(2C_{0}\right)}^{2}}-C_{x}W}\right)$$

where *W* $\equiv \left(\omega_{K}-\omega_{\mathrm{Cl}}\right)/\left(\omega_{K}+\omega_{\mathrm{Cl}}\right)$; $\omega_{K}\;$ and $\omega_{\mathrm{Cl}}$ are the mobility of K^+^ and Cl^−^ ions, respectively, in the membrane; and *F*, *R*, and *T* are the Faraday constant, gas constant, and absolute temperature, respectively. 

### 2.8. Measurement of Membrane Resistance

The electrical resistances of the membranes immersed in 0.5 mol/dm^3^ NaCl were measured using an acrylic plastic cell with an effective measurement area of 1.0 cm^2^, as described elsewhere [1,41]. During the measurement, 10 kHz AC was applied to Pt electrodes inside the cell. First, the electrical resistance *R*_o_ of the 0.5 mol/dm^3^ NaCl solution was measured at 25.0°C ± 0.5°C. Subsequently, a sample membrane was set in the cell, and the resistance *R*_s_ was measured. The membrane resistance *R*_m_ was calculated by subtracting *R*_o_ from *R*_s_.

### 2.9. Measurement of Dynamic State Transport Number

**Figure 2:**
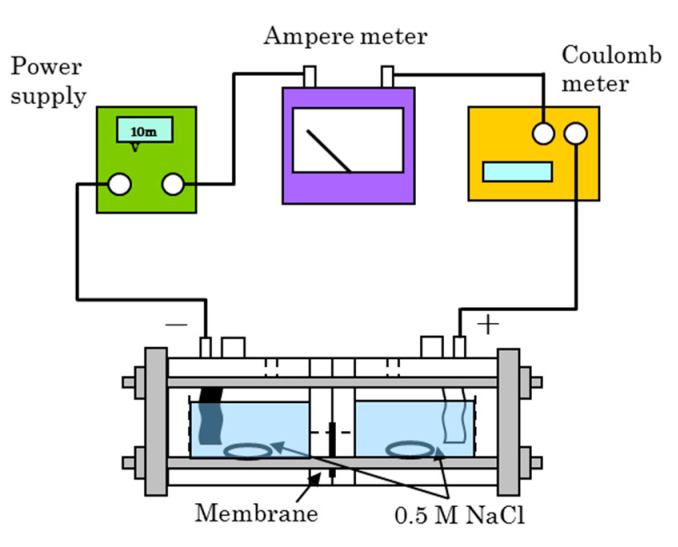
Apparatus for the electrodialysis experiments. The effective area of the cell was 4.0 cm^2^.

The dynamic state transport number *t*_d+_ of a sample membrane was determined via electrodialysis (ED) performed using a cell with two chambers separated by a membrane (Figure 2). Direct current was applied between the two electrodes (Ag–AgCl) of the cell containing 0.5 mol/dm^3^ NaCl in the two chambers at a current density of 10 mA/cm^2^ at 25 °C [1]. The conductivity change across the two chambers during ED was measured in order to obtain the equivalent change due to ion transport through the membrane. The dynamic state transport number was obtained in terms of the equation:

(4) $$t_{d+}=\frac{\Delta mVF}{Q},$$

where Δ*m*, *V*, and *Q* are the equivalent change of ions, volume of the solution, and electric charge passing through the membrane during the ED test, respectively.

### 2.10. Measurement of Mechanical Strength 

The mechanical strength, tensile strength, Young’s modulus, and elongation at the breaking point of the wet membranes were measured using a SHIMADZU EZ-Test50N. Specimens with a nominal 20-mm gauge length and 2-mm gauge width were punched out using a dumbbell-type punch. Three specimens were tested at a test speed of 20 mm min^−1^, and the average values were reported.

## 3. Results and Discussion

### 3.1. Characterization of Synthesized Polymer

**Figure 3:**
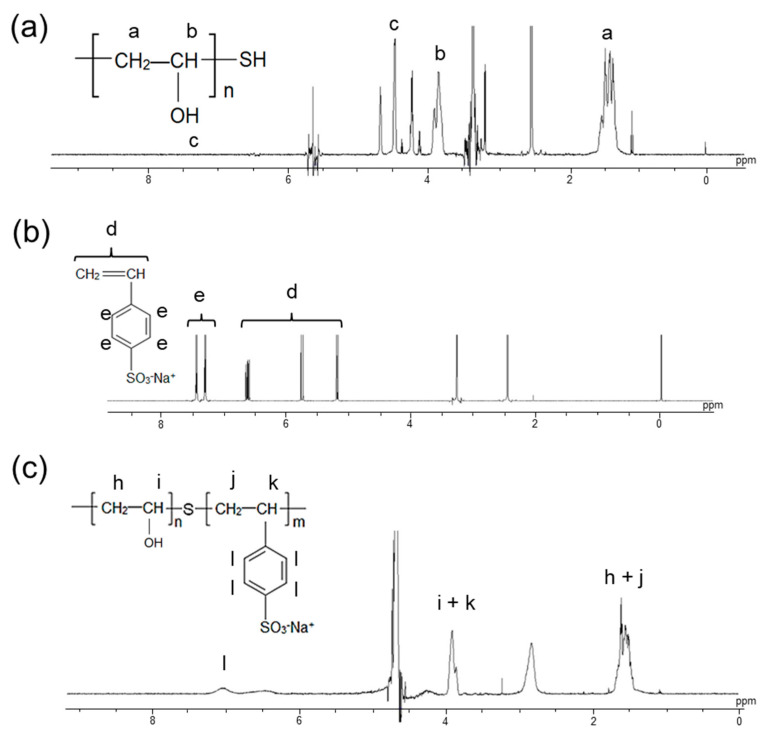
^1^H NMR spectra of (**a**) PVA-SH, (**b**) SSS monomer, and (**c**) PVA-*b*-PSSS graft copolymer in DMSO-d_6_.

Figure 3a–c shows the ^1^H NMR (DMSO-d_6_) spectra of PVA-SH, SSS monomer, and synthesized block copolymer PVA-*b*-PSSS, respectively. New peaks corresponding to the aromatic protons of the SSS groups appeared between 7.0 and 8.0 ppm in the spectra of PVA-*b*-PSSS, indicating that the desired block copolymer was successfully synthesized. The content of SSS groups in the copolymer was defined as the molar ratio of SSS groups to PVA-*b*-PSSS main chains, which is the same meaning as that of cation-exchange groups in the copolymer *C*_CEG_. The value was calculated via the integrals of the aromatic protons of SSS groups at 7.0–8.0 ppm, and those of the methylene groups at 1.2–1.6 ppm. Table 1 shows the reaction conditions, SSS monomer content C^m^_SSS_, and *C*_CEG_ in the graft copolymer calculated from the ^1^H NMR spectra. *C*_CEG_ varied from 2.91 to 10.7 mol%, and was essentially proportional to C^m^_SSS_. The value of *C*_CEG_ was approximately 20% lower than that of C^m^_SSS_, which may be attributed to incomplete polymerization of the monomer, or to the formation of a homopolymer—poly(SSS)—which was dissolved into acetone during the precipitation process and into the aqueous solution during the crosslinking process. 

### 3.2. Ion-Exchange Capacity and Water Content of B-CEMs as a Function of SSS Content

**Figure 4:**
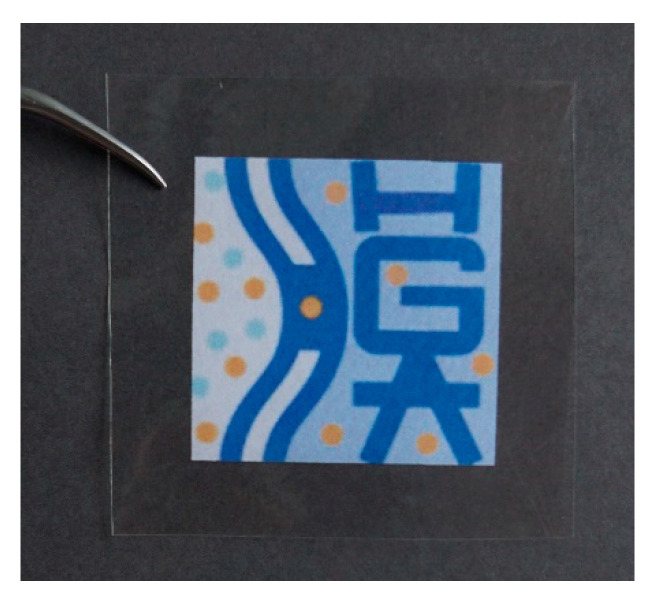
Photograph of one of the prepared B-CEMs.

Figure 4 shows one of the prepared CEMs from PVA-b-PSSS. The CEM was transparent, and had a self-standing membrane even without any supporting material. The preparation conditions, water contents *H*, and *IECs* of the B-CEMs are listed in Table 2. CEMs with high *IEC* values are desirable for the preparation of high-performance IEMs with high permselectivity for cations and low membrane resistance. Conversely, these high *IEC* values may also result in a degradation of the mechanical properties of the CEMs, because the membrane water content increases with increasing *IEC*. Therefore, controlling the amount of sulfonate groups in the block copolymers is essential. The *IEC* values of the B-CEMs were directly proportional to the SSS content of the copolymers, demonstrating that the *IEC* values can be estimated from the SSS contents of the copolymers. By comparison, the *IEC* of a commercially available CEM—Neosepta^®^ CMX (ASTOM Corp., Japan)—is 2.0 meq/g-dry. Hence, the *IEC* of B-CEM-10, B-CEM-11, and B-CEM-12 (10.7 mol% of *C*_CEG_) is 70% that of CMX. 

**Figure 5:**
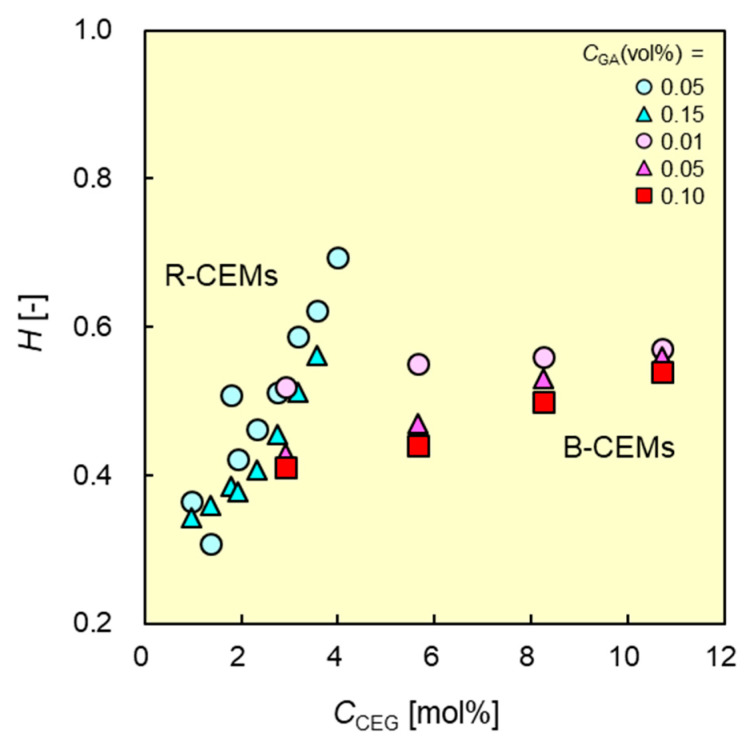
Water contents *H* of B-CEMs and R-CEMs as functions of content $C_{\mathrm{CEG}}$ of cation-exchange groups in copolymers. Red and blue symbols denote the data of B-CEMs and R-CEMs, respectively.

Figure 5 shows the water contents of the CEMs as functions of *C*_CEG_. To compare the characteristics between B-CEMs and R-CEMs, the data indicated by the blue symbols depict the R-CEMs from a previous study [37]. The water content of both types of IEMs increased with increasing *C*_CEG_, owing to the increased the number of charged groups in the CEM, resulting in an increase in the difference between the osmotic pressure inside the membrane and that of the solution in which the membrane was immersed. The CEMs crosslinked with high concentrations of GA had low water contents because the chemical crosslinking with GA increases the number of chemical crosslinking points in the membrane. The B-CEMs had lower water contents than the R-CEMs at the same molar percentage of the cation-exchange groups. For example, the water contents of B-CEM-2 (*C*_CEG_ = 2.9 mol%; *C*_GA_ = 0.05 vol.%) and an R-CEM (*C*_CEG_ = 3.1 mol% and *C*_GA_ = 0.05 vol.%) were 0.43 and 0.59, respectively; the difference can be attributed to the higher crystallinity of the PVA region in the B-CEM compared to that in the R-CEM. Therefore, it will be difficult to prepare R-CEMs with a *C*_CEG_ greater than 6 mol% because the CEMs will have water contents greater than 0.8 and, thus, their mechanical strengths will be insufficient to support self-standing membranes.

### 3.3. Membrane Charge Density as a Function of $C_{CEG}$

The membrane charge density is defined as the ratio of the molarity of the fixed charged groups to the water volume in the membrane; hence, it is proportional to the ratio of the IEC to the water content of a CEM as:

(5) $$C_{x}\propto \frac{IEC}{H}$$

**Figure 6:**
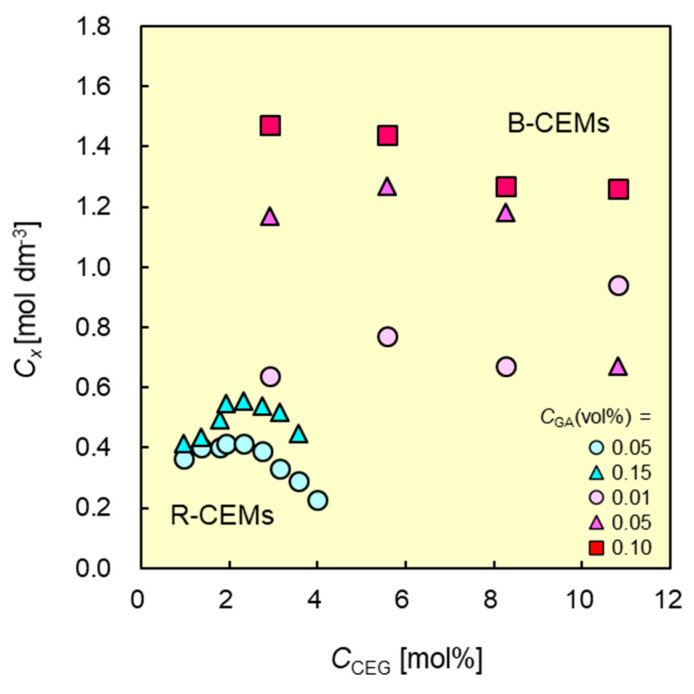
Charge densities *C*_x_ of B-CEMs and R-CEMs as functions of contents $C_{\mathrm{CEG}}$ of cation-exchange groups in copolymers. Red and blue symbols denote the data of B-CEMs and R-CEMs, respectively.

The charge density affects the selective cation transport of a CEM. A CEM with high charge density will have a high counter-ion permselectivity. The charge density of both CEMs tends to increase with *C*_CEG_, reaching a maximum value (Figure 6). This is because the *IEC* is directly proportional to the *C*_CEG_ (Table 2). In addition, *H* also increased with increasing *C*_CEG_ (Figure 5). From these points, we can conclude that the charge density initially increased with increasing *C*_CEG_ because the *IEC* increased. At high *C*_CEG_ values, the increase in water content has a greater effect on the charge density than the increase in the *IEC*. Hence, the charge density decreased after reaching a maximum value. The maximum value of the charge density increased with increasing *C*_GA_ because *H* decreased with increasing *C*_GA_ (Figure 5). B-CEM-3 (*C*_CEG_ = 2.9 mol%; *C*_GA_ = 0.10 vol.%) had a maximum value of 1.47 mol/dm^3^, whereas that of CMX was 1.8 mol/dm^3^. This indicates that the B-CEM had almost 80% of the charge density of the commercially available CEM. Of all tested CEMs, the B-CEMs exhibited much higher charge densities than the R-CEMs at all tested *C*_GA_ values. For example, the charge density of B-CEM-2 (*C*_CEG_ = 2.9 mol%; *C*_GA_ = 0.05 vol.%) indicated 1.2 mol/dm^3^, while that of an R-CEM (*C*_CEG_ = 3.1 mol%; *C*_GA_ = 0.05 vol.%) was 0.33 mol/dm^3^. The charge density of the B-CEM was higher than that of the R-CEM, even when the two CEMs had essentially the same *C*_CEG_, owing to the lower water content of the former (Figure 5).

### 3.4. Membrane Resistance as Function of Water Content

**Figure 7:**
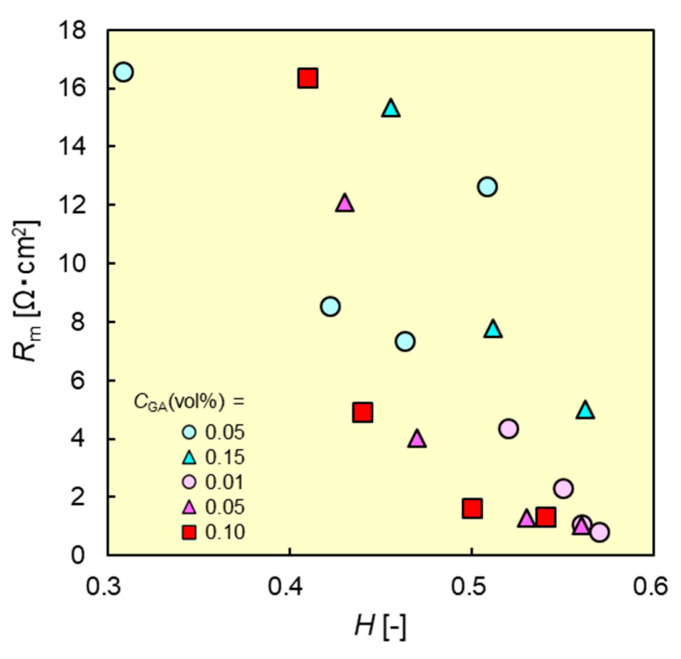
Membrane resistances *R*_m_ of B-CEMs and R-CEMs as functions of water content *H*. Red and blue symbols denote the data of B-CEMs and R-CEMs, respectively.

Membrane resistance affects the energy efficiency of the electrodialysis process. Figure 7 shows the resistance of PVA-based CEMs as functions of *H*. The resistance of the fabricated CEMs decreased as *C*_CEG_ increased, because CEMs with high *C*_CEG_ had both a high water content and a high *IEC*. By contrast, the resistance increased with increasing *C*_GA_. This result implies that the membrane resistance can be controlled by altering the water content of the CEMs. Of all of the CEMs, B-CEM-10 (*C*_CEG_ = 10.7 mol%; *C*_GA_ = 0.01 vol.%) exhibited the lowest membrane resistance of 0.81 Ω cm^2^. 

### 3.5. Dynamic State Transport Number of CEMs vs. C_CEG_

**Figure 8:**
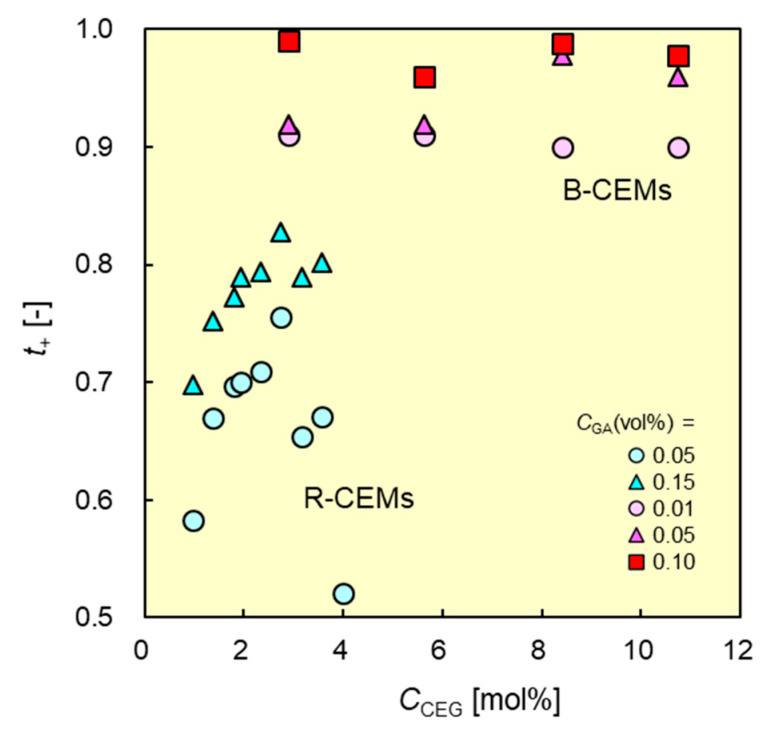
Dynamic state transport numbers *t*_+_ of B-CEMs and R-CEMs vs. $C_{\mathrm{CEG}}$ of cation-exchange groups in copolymers. Red and blue symbols denote the data of B-CEMs and R-CEMs, respectively.

The cation permselectivity of a CEM in an ED process can be expressed by dynamic state transport number. An ideal CEM is indicated by a cationic transport number of 1.0, meaning that the CEM allows only the permeation of cations in an ED system. The transport numbers of the B-CEMs were more than 0.90, almost independent of *C*_CEG_ (Figure 8). By contrast, a CEM crosslinked with high *C*_GA_ showed the highest transport number_._ These results indicate that the transport numbers of the CEMs can be controlled via changes in the concentration of the crosslinking agent, which in turn affects the degree of crosslinking. The B-CEMs with essentially the same *C*_CEG_ and *C*_GA_ values exhibited higher transport numbers than the R-CEMs. For example, B-CEM-2 (*C*_CEG_ = 2.9 mol%; *C*_GA_ = 0.05 vol.%) had a transport number of 0.92, whereas that of an R-CEM (*C*_CEG_ = 3.1 mol% and *C*_GA_ = 0.05 vol.%) was 0.65, 1.42 times lower than that of the B-CEM. The B-CEMs had essentially the same dynamic transport numbers as the commercial CEMs. For example, B-CEM-9 (*C*_CEG_ = 8.3 mol%; *C*_GA_ = 0.10 vol.%) exhibited a transport number of 0.99, whereas that of CMX was 0.98 under the same conditions.

### 3.6. Relationship between Membrane Resistance and Dynamic State Transport Number

**Figure 9:**
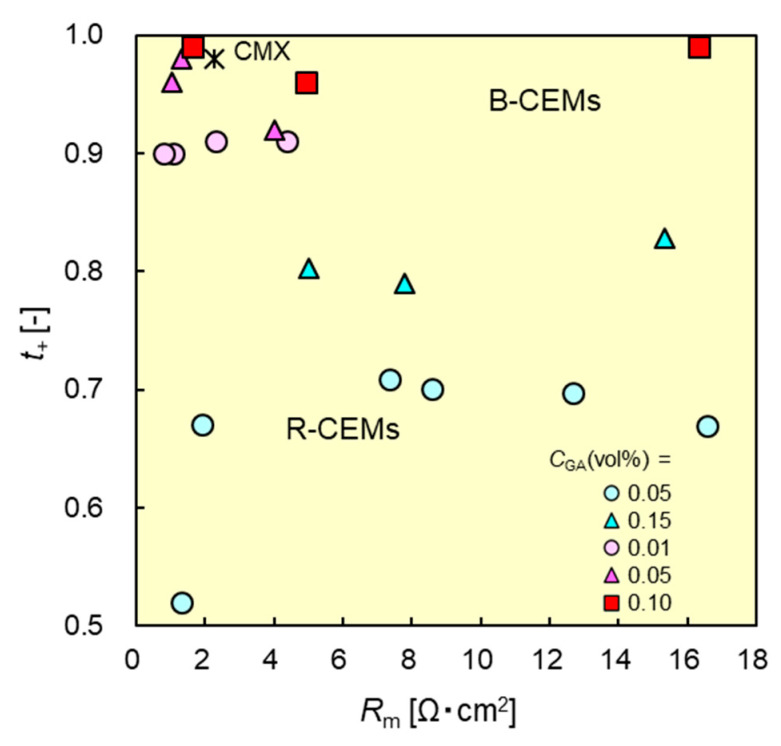
Relationship between dynamic state transport numbers *t*_d+_ and membrane resistance *R*_m_ of B-CEMs and R-CEMs. Red and blue symbols denote the data of B-CEMs and R-CEMs, respectively. *: The data of the commercial CEM (CMX).

An ion-exchange membrane with both high ion permselectivity and low membrane resistance is desirable in IEM applications such as electrodialysis (ED), reverse electrodialysis (RED), fuel cells, and redox flow batteries. Figure 9 shows the relationship between the dynamic state transport numbers and the resistances of the CEMs. The CEM located at the upper left-hand side of the figure exhibited high performance in the aforementioned applications. B-CEMs typically exhibited higher transport numbers than R-CEMs with similar membrane resistances. For example, B-CEM-9 with a resistance of 1.6 Ωcm^2^ exhibited a transport number of 0.99, whereas that of the R-CEM with almost the same resistance (1.9 Ωcm^2^) was 0.67; thus, the transport number of the B-CEM was approximately 48% higher than that of the R-CEM, although both CEMs had similar membrane resistances. The dynamic transport number and membrane resistance of B-CEM-9 were 0.99 and 1.6 Ωcm^2^, respectively, whereas those of CMX were 0.98 and 2.3 Ωcm^2^, respectively. The B-CEM prepared herein had almost the same dynamic transport number, whereas its membrane resistance was 70% that of the commercially available CEM. 

### 3.7. Mechanical Strength of B-CEMs

The mechanical properties of the B-CEMs are listed in Table 3. Here, B-CEM-12 was too brittle to measure its mechanical strength. The tensile strengths of the B-CEMs were lower than that of the CMX membrane, because the B-CEMs did not have any support materials, whereas the commercial ion-exchange membranes were supported by materials such as PVC cloth [42]. Hence, PVA-based CEMs fabricated with support materials are expected to have sufficient mechanical strength for application in electrodialysis desalination processes at low salt concentrations. 

## 4. Conclusions

In this study, CEMs with IPN structures were fabricated from a PVA-based block copolymer—PVA-*b*-PSSS—containing various molar percentages of cation-exchange groups, followed by crosslinking with various concentrations of crosslinking agent. The B-CEMs thus obtained had lower water content than R-CEMs with the same molar percentages of cation-exchange groups.

The charge densities of the membranes increased with increasing *C*_CEG_ and increasing *C*_GA_. A maximum charge density of 1.47 mol/dm^3^ was obtained with B-CEM-3 (*C*_CEG_ = 2.9 mol%; *C*_GA_ = 0.10 vol.%), which was almost two-thirds of the charge density of CMX—a commercially available CEM.

The B-CEMs exhibited higher transport numbers than the R-CEMs with similar membrane resistances. The dynamic transport number and membrane resistance of B-CEM-9 (*C*_CEG_ = 8.3 mol%; *C*_GA_ = 0.10 vol.%) were 0.99 and 1.6 Ωcm^2^, respectively, whereas those of CMX were 0.98 and 2.3 Ωcm^2^, respectively. 

Optimization of the molar ratio of the cation-exchange groups to vinyl alcohol groups, and of the physical and chemical crosslinking conditions, (particularly the annealing temperature and crosslinker concentration), reduced the membrane resistance of PVA-based CEMs. With high counter-ion permselectivities and low membrane resistances, the developed PVA-based CEMs exhibit a similar performance in ED and RED processes to that of commercially available CEMs. Furthermore, the PVA-based block copolymer can prepare a self-standing ion-exchange layer, indicating that the ion-exchange layer has higher mechanical strength than the ion-exchange domain of styrene-divinylbenzene-based matrix that is used in commercial IEMs. Therefore, a PVA-based CEM can be manufactured by coating the polymer solution on non-woven fabric support. The CEMs manufactured in the process can potentially have a lower cost than commercial CEMs, and are thus expected to be even more cost-effective and efficient in applications involving ED and RED systems. 

## 5. Research Highlights

➢ Block-type, PVA-based cation-exchange membranes (B-CEMs) were prepared.
➢ The transport numbers of B-CEMs were higher than those of R-CEMs.
➢ B-CEMs exhibit similar transport numbers to a commercial CEM.

## Figures and Tables

**Table 1:** Reaction conditions of PVA-*b*-PSSS block copolymers.

Sample	PVA [g]	SSS [g]	H_2_O [g]	V-50 [g]	*C*^m^_SSS_ [mol%]	*C*_CEG_ [mol%]
PVA-*b*-PSSS-1	35.6	8.00	230	0.14	4	2.91
PVA-*b*-PSSS-2	35.6	13.0	250	0.22	7	5.66
PVA-*b*-PSSS-3	35.6	20.0	273	0.34	10	8.25
PVA-*b*-PSSS-4	28.5	20.8	232	0.35	13	10.7

V-50: polymerization initiator; *C*^m^_SSS_: SSS content in reaction mixture; *C*_CEG_: the cation-exchange group content in the obtained copolymer.

**Table 2:** Cation-exchange group contents *C*_CEG_, GA concentrations *C*_GA_ for different preparation conditions, thicknesses *d*_W_ of the wet membranes, water content *H*, and ion-exchange capacities *IEC* of B-CEMs.

Sample	*C*_CEG_ [mol%]	*C*_GA_ [vol. %]	*d*_W_ [µm]	*H* [-]	*IEC* [meq/g]
B-CEM-1	2.91	0.01	76	0.52	0.56
B-CEM-2		0.05	82	0.43	
B-CEM-3		0.10	72	0.41	
B-CEM-4	5.66	0.01	97	0.55	0.79
B-CEM-5		0.05	92	0.47	
B-CEM-6		0.10	90	0.44	
B-CEM-7	8.25	0.01	89	0.56	1.05
B-CEM-8		0.05	85	0.53	
B-CEM-9		0.10	112	0.50	
B-CEM-10	10.7	0.01	109	0.57	1.38
B-CEM-11		0.05	119	0.56	
B-CEM-12		0.10	101	0.54	

**Table 3:** Maximum tensile strengths (*TS*), Young’s moduli (*YM*), and elongations at breaking point (*E*) of B-CEMs and CMX (a commercial CEM). The properties of B-CEM-12 could not be measured, owing to its brittleness.

Sample	*TS* [MPa]	*YM* [MPa]	*E* [%]
B-CEM-1	4.98	28.2	17.7
B-CEM-2	1.48	46.3	4.98
B-CEM-3	5.14	74.4	1.66
B-CEM-4	3.58	77.9	11.0
B-CEM-5	2.10	39.2	2.18
B-CEM-6	4.63	71.0	2.24
B-CEM-7	3.86	63.6	10.9
B-CEM-8	6.13	50.7	4.90
B-CEM-9	2.38	35.6	3.07
B-CEM-10	2.33	6.43	8.80
B-CEM-11	7.75	41.7	3.57
B-CEM-12	-	-	-
CMX	36.7	1120	14.1

## Data Availability

All data generated or analysed during this study are included in this published article.
